# A Genomic Characterization of Clinical *Brucella melitensis* Isolates from Tunisia: Integration into the Global Population Structure

**DOI:** 10.3390/microorganisms13020243

**Published:** 2025-01-23

**Authors:** Asma Ferjani, Hellen Buijze, Germán Kopprio, Susanne Köhler, Amel Rehaiem, Hajer Battikh, Lamia Ammari, Sana Ferjani, Lamia Kanzari, Meriam Zribi, Badreddine Kilani, Nicolle Hanschmann, Holger Scholz, Ilhem Boutiba

**Affiliations:** 1 Laboratory of Microbiology, Charles Nicolle Hospital, Tunis 1006, Tunisia; rehaiem_amel@yahoo.fr (A.R.); ferjanisana.sa@gmail.com (S.F.); lamiakanzari1@gmail.com (L.K.); ilhemboutiba@gmail.com (I.B.); 2 Research Laboratory Antimicrobial Resistance LR99ES09, Faculty of Medicine of Tunis, University of Tunis El Manar, Tunis 1007, Tunisia; 3 Department of Microbiology, Universitätsklinikum Augsburg, 86156 Augsburg, Germany; hellen.buijze@uk-augsburg.de; 4 Highly Pathogenic Microorganisms, Centre for Biological Threats and Special Pathogens (ZBS2), Robert Koch Institute, 13353 Berlin, Germany; koppriog@rki.de (G.K.); koehlers@rki.de (S.K.); hanschmannn@rki.de (N.H.); scholzh@rki.de (H.S.); 5 Laboratory of Microbiology, Rabta Hospital, Tunis 1006, Tunisia; hajerbattikh@yahoo.fr (H.B.); meriamzribimiled@gmail.com (M.Z.); 6 Infectious Disease Department, Rabta Hospital, Tunis 1006, Tunisia; lamia_ammarib@yahoo.fr (L.A.); badreddinekilani@yahoo.fr (B.K.)

**Keywords:** *Brucella melitensis*, Tunisia, whole-genome sequencing, core genome multilocus sequence typing (cg MLST)

## Abstract

Brucellosis represents a significant global health concern that is endemic in many regions of the world, especially in Maghreb (Tunisia, Morocco and Algeria). In Tunisia the diagnosis of human brucellosis is primarily based on serological tests and cultivation of the causative pathogen, without the knowledge of the underlying *Brucella* species or biovar. In addition, the scarcity of laboratories with adequate biosecurity measures to handle suspected specimens constitutes a significant challenge. Furthermore, the absence of full genome data limits our understanding of the genetic diversity of *Brucella* in Tunisia in comparison to the strains circulating in the North African region and the world. In the present study, a total of 36 bacterial isolates derived from human patients diagnosed with brucellosis in Tunisia were subjected to examination. Real-time PCR confirmed all isolates as *B. melitensis*. In the subsequent core genome-based MLST analysis (cgMLST) based on 2706 target genes, the isolates formed two separate but closely related clusters with a distance of 140 alleles. The intra-cluster diversity was one to six alleles. In the larger geographical context and in comparison to almost 1000 other *Brucella* genomes, the isolates showed the highest genetic relationship to *B. melitensis* isolates from Italy and Egypt with distances of 130 and 150 alleles, respectively. All the isolates were most similar to the biovar 3 genotype. Markedly, strains from a reported brucellosis outbreak in Austria were grouped closely (26 and 27 alleles, respectively) together with strains from Tunisia, suggesting that this country may represent their geographical origin. This research represents a significant advancement in our understanding of *B. melitensis* strains circulating in the Maghreb region, as it is the first study to elucidate the molecular characterization of strains isolated from humans in Tunisia. The cgMLST analysis of the strains provided information on the regional distribution of the strains and the association with neighboring countries and significant outbreaks in the region. The data will form the basis of a future reference framework for strains circulating in the Mediterranean region.

## 1. Introduction

Human brucellosis, a disease caused by the *Brucella* species of bacteria, is one of the most prevalent zoonotic diseases globally, with approximately 500,000 new cases occurring every year [1]. The epidemiology of this disease has acutely changed over the past decade because of various sanitary, socioeconomic and political reasons [2]. Despite the availability of control measures such as animal vaccination, surveillance of abortions and pasteurization of milk products, brucellosis remains endemic in many countries of the Mediterranean region, the Middle East, Southern Europe, Central Asia, the Indian subcontinent and Africa [1,3]. In 2017, the overall incidence in Tunisia was 9.8 per 100,000 population, with 621 new human cases in the mentioned year [4].

Humans represent an occasional host infected via direct contact with infected goats or sheep or infected indirectly through the consumption of raw meat and unpasteurized milk and dairy products contaminated with *Brucella* [5]. Therefore, brucellosis represents a significant public health concern as well as an economic burden, as compromised food security requires counteractive measures (quarantine or slaughter) with critical impact on livestock productivity [1]. Human-to-human transition of *Brucella* is rare; it can occur transplacentally, via breastfeeding and rarely through sexual intercourse, organ transplantation and blood [3]. The *Brucella* genus is considered one of the most common pathogens causing laboratory-acquired infections (LAIs) which are an increasingly important biosafety issue [6]. *Brucella* species are also considered potential bioterrorism agents [7]. If acquired and adequately diffused, *Brucella* could cause a serious public health challenge in terms of the ability to limit fatalities and control the other consequences of such an attack [8].

The genus *Brucella* comprises more than 12 species with a percentage of similarity close to 100% through DNA–DNA hybridization [9,10]. Of these, *B. melitensis* is the predominant species, causing the majority of human cases [11,12,13]. Despite their close genetic relationship, *Brucella* species exhibit a wide variety of phenotypic characteristics, pathogenicity and host preferences, thus requiring powerful universal diagnostic tools. A high difference between the reported rate and the actual incidence rate has been reported, largely due to misdiagnosis and underdiagnosis, especially in endemic areas [14]. Currently, the diagnosis of human brucellosis in Tunisia is based on a combination of clinical examination, serological tests, cultivation and bacterial isolation from biological samples. These methods are generally time-consuming and require laboratory-qualified personnel, as well as a biosafety level 3 facility [15,16]. If laboratories are equipped for it, real-time PCR-based assays are the method of choice for the rapid and reliable diagnosis of brucellosis. The assays currently in use focus on identifying different species of the genus *Brucella* based on its highly conserved genetic loci (e.g., *BCSP 31*, *IS6501/711* or 16S rRNA genes).

Core genome multilocus sequence typing (cgMLST) is widely used to assess genetic relatedness among bacterial isolates to support outbreak investigation [17,18] or trace back transmission chains [19]. Whole-genome sequencing (WGS)-based phylogeny stands as the most powerful tool to discriminate *Brucella* strain identity, origin and their phylogenetic relationship [20]. This approach facilitates a comprehensive understanding of the global epidemic.

However, the lack of epidemiological data from Tunisia and its neighboring countries represents a significant obstacle to the assessment of the clinical impact in this region. The objective of this study was twofold: Firstly, to examine the classification, within the context of both the local epidemiological landscape and the global epidemiological picture, of strains identified in Tunisia through the use of whole-genome sequencing. And, secondly, to describe the virulence patterns of these clinical *Brucella melitensis* strains isolated in Tunisia.

## 2. Materials and Methods

### 2.1. Isolation of Bacteria

**Figure 1:**
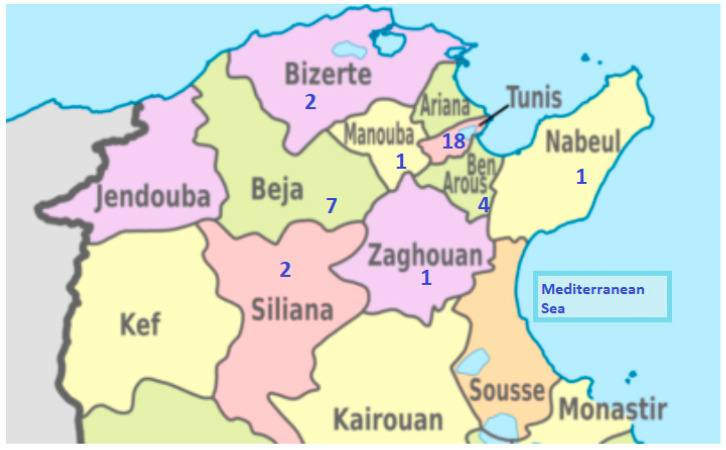
The distribution of *B. melitensis* strains collected from Tunisian infected humans in the 2015–2017 period.The figure indicates the number of *B. melitensis* strains isolated in each of eight governorates: Nabeul (1), Mannouba (1), Zaghouan (1), Bizertz (2), Tunis (18), Beja (7) and Seliana (2).

A total of 36 strains of suspected *Brucella* spp. were obtained from 36 blood cultures collected between January 2016 and December 2020 from patients coming from different regions of Tunisia (Figure 1). They were suspected of acute brucellosis and hospitalized in the infectious diseases departments of two university hospitals in Tunisia: Hôpital Charles Nicolle (the biggest and the oldest hospital in Tunisia) and Hôpital La Rabta, the latter being the reference center of infectious diseases responsible for handling patients in need coming from different northern regions. Blood samples were placed in an automatic incubator (Bactalert 3D, BioMérieux, Marnes La Coquette, France). Positive blood cultures were plated on Columbia blood agar (BioMérieux, France) containing 5% sheep blood and incubated for 48 h at 36 ± 1 °C with 5% CO_2_. All strains were studied for colony morphology and culture features and were characterized by morphological (e.g., microscopic stain) and biochemical tests such as oxidase, catalase, urease and Api 20E gallery identification (BioMérieux, Marnes La Coquette, France). Strains identified as *Brucella* spp. were stored at −80 °C in Brain Heart Infusion (BHI) with 15% glycerol.

### 2.2. Identification of Bacterial Strains Through Real-Time PCR

Total DNA was extracted from single colonies of subcultures using a DNeasy Blood and Tissue Kit (Qiagen, Hilden, Germany) following the manufacturer’s protocol for Gram-negative bacteria. For the multiplex PCR (Table 1), two *Brucella* gene markers were targeted (IS711 and MazG), as well as an internal positive control (IPC). The IPC was a chemically synthetized sequence (Koma2) cloned into the pPCR-Script Vector (Stratagene, LA Jolla CA). It was added to the samples before extraction at a concentration of 250 copies per sample. Detection of its presence at an average Ct value of 35 ± 3 within the multiplex PCR verified the success of the extraction and the functionality of the PCR set up. The localization of the primers and probes for *Brucella* detection was based on the sequences of the MazG gene (GenBank U78089) and the IS711 gene (GenBank M94960). PCR amplification was performed in a final volume of 25 µL. The reaction mixtures consisted of 5 µL of DNA template, 6.25 µL of TaqMan Environmental Master Mix 2.0 (Life Technologies, Carlsbad, CA, USA), 0.75 µL of each primer (10 µM), 0.25 µL of probe (10 µM) and 8.5 µL of free nuclease water (Fluka, Lincolnshire, IL, USA).

For amplification, an ABI 7500 system (Applied Biosystems, Waltham, MA, USA) was used following a standard amplification program (Table 2). DNA from the reference strain of *Brucella melitensis* bv. 1 str. 16M was used as a positive control. The concentration of this DNA was adjusted to 0.2 pg µL^−1^ by dilution with AE buffer (Qiagen, Hilden, Germany) so that a Ct value between 31 and 35 (depending on the target marker) was obtained by real-time PCR.

### 2.3. Whole-Genome Sequencing and Annotations

Total genomic DNA was quantified with the Qubit fluorometer (Thermo Fisher Scientific, Waltham, MA, USA), and library preparation was performed using the Nextera XT library preparation kit (Illumina, San Diego, CA, USA). The libraries were sequenced using the Illumina NextSeq 500 platform, producing 150 bp paired-end reads. After demultiplexing and removal of adapters, reads were trimmed and filtered with fastpv0.22.0 using default options [21]. Quality control of raw and filtered reads was conducted with FastQCv0.11.9 [22]. The de novo whole-genome assemblies were performed with SPAdesv3.15.5 using the option –careful [23]. Contigs with a length under 1000 bp were removed with bbmap v39.01. The species *Brucella melitensis* and the percentages of completeness and contamination were inspected with CheckM v1.2.2 [24]. Virulence and antimicrobial resistance (AMR) genes in all Tunisian strains and two reference strains were detected using ABRicate v1.0.1 with the databases VFDB and MEGARes, and AMR Finder Plus3.11.4 with the NCBI database and the option –plus.

### 2.4. Core Genome Multilocus Sequence Typing (cgMLST)

A cgMLST scheme for *B. melitensis* with 2704 targets in the core genome and 360 targets in the accessory genome was used [25]. Basically, a genome-wide gene-by-gene comparison using the cgMLST Target Definer (version 1.4) function of the SeqSphere software, v5.0.90 (Ridom GmbH, Münster, Germany), was performed. Default parameters and the following filters were selected to exclude certain genes of the *B. melitensis* bv. 1 strain 16M reference genome (NC_003317.1 and NC_003318.1) from the cgMLST scheme: a minimum length filter that discarded all genes shorter than 50 bp, a start codon filter that discarded all genes that contain no start codon at the beginning of the gene, a stop codon filter that discarded all genes that contain no stop codon or more than one stop codon or if the stop codon is not at the end of the gene, a homologous gene filter that discarded all genes with fragments that occur in multiple copies within a genome (with identity of 90% and more than 100 bp overlap) and a gene overlap filter that discarded the shorter gene from the cgMLST scheme if the two genes affected overlap by 4 bp. The remaining genes were then used in a pairwise comparison using BLAST (version 2.2.12), with the query chromosomes of one representative for each of the other two *B. melitensis* biovars (*B. melitensis* bv. 2 strain 63/9 [NZ_CP007788.1 and NZ_CP007789.1] and *B. melitensis* bv. 3 strain Ether [NZ_CP007761.1 and NZ_CP007760.1]). Using all genes of the reference genome that were common in all query genomes, with a sequence identity of 90% and 100% overlap, and with the genome filters start codon filter, stop codon filter and stop codon percentage filter turned on, the final cgMLST scheme was formed. Therefore, all genes that had no start or stop codon in one of the query genomes, as well as genes that had internal stop codons in more than 20% of the query genomes, were discarded. An additional cgMLST was performed using the pipeline and database of PubMLST for *Brucella* species [26]. The cgMLST scheme from Tunisian strains was obtained using the Genome Compartor tool in PubMLST.

### 2.5. Clustering Analyses, Cluster Definition and Data Availability

The cgMLST profiles were assigned using the *B. melitensis* task template in SeqSphere. Minimum spanning trees (MSTs) were created by pairwise comparison of cgMLST target genes using default software settings. Missing values were ignored in the calculation of the distance between pairs of sample profiles. The links between the MST nodes represented the distance between the genotypes. The cluster cutoff value was defined as the maximum pairwise distance found between epidemiologically related isolates. In the case of PubMLST, MSTs were created with GrapeTree using default parameters [27].

All generated data were submitted to the National Center for Biotechnology Information (NCBI) under the Bioproject IDPRJNA1053259. The 36 assemblies were assigned - accession numbers from SAMN38851271 to SAMN38851306 (see Table 3 for further details).

### 2.6. Acquisition of Metadata

Demographic and clinical data were obtained from human brucellosis cases infected by the isolated strains. Physicians from different departments were contacted to complete an information sheet for each patient including the following: origin, age, gender and symptoms (see Table 3).

## 3. Results

### 3.1. Collection of Isolates and Molecular Identification

The respective demographic and clinical data for the 36 brucellosis cases are summarized in Table 3. The real-time PCR assays performed in parallel at the Robert Koch Institute (RKI) in Berlin (Germany) and at Charles Nicole Hospital in Tunis (Tunisia) yielded identical results: all isolates were identified as *B. melitensis*.

### 3.2. Whole-Genome Analysis and Core Genome Multilocus Sequence Typing (cgMLST)

**Figure 2:**
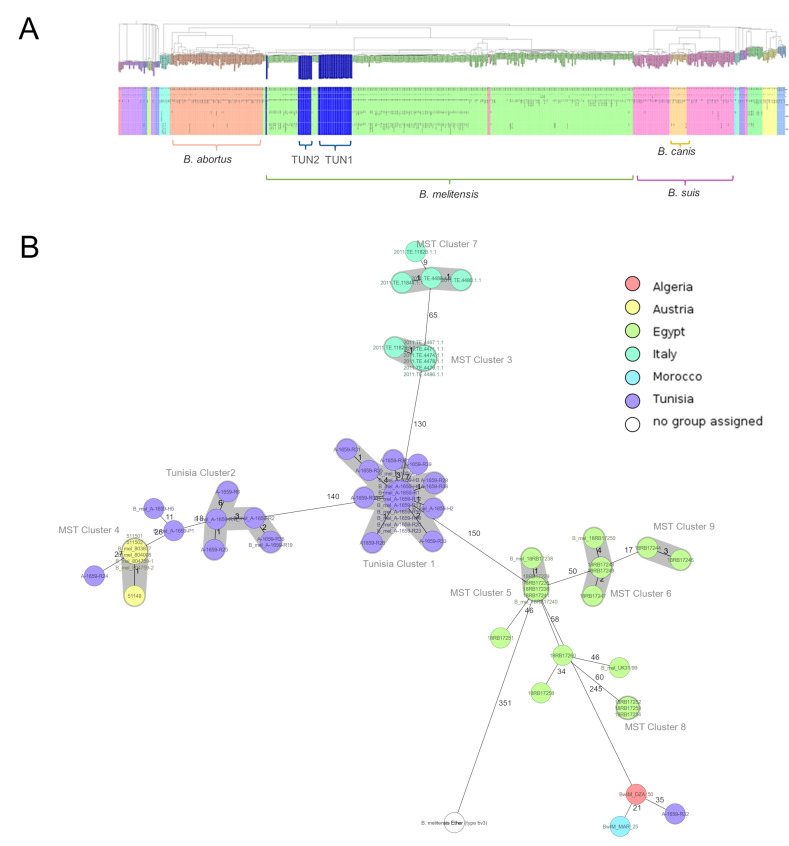
Core genome Multilocus Sequence Typing (cgMLST)-based population trees of *Brucella* isolates. (**A**) Neighbor-joining tree of >1000 *Brucella* spp. isolates revealing two genetically close clusters of Tunisian *B. melitensis* isolates within global population structure. (**B**) Minimum spanning tree of 36 Tunisian *B. melitensis* isolates and their closest relatives color-coded by geographic origin. Distance labels correspond to number of discriminating alleles.

All cultivable isolates were subjected to whole-genome sequencing. All assemblies yielded an average coverage of ~130× and corresponded to *B. melitensis* with over 99% completeness and less than 1.3% contamination. All Tunisian isolates cluster together with *B. melitensis* biovar 3 (Figure 2).

**Figure 3:**
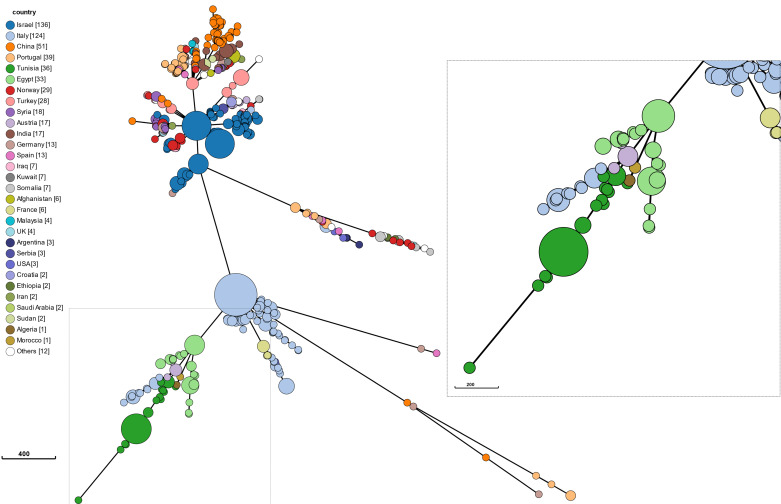
Minimum Spanning Tree (MST) generated for 36 Tunisian strains including all databases worldwide in PubMLST. The number after the country in the brackets indicates the number of strains.

Both tested schemes used to determine genetic relatedness between the isolates were comparable and revealed identical groupings. with only few-allele differences between the two assays. Within the global population structure, Tunisian isolates form two distinct clusters with a distance of 140 discriminating alleles. Within the proposed clusters, strains differed in up to 6 alleles, with 14 isolates from the proposed Tunisia Cluster 1 showing not a single discriminatory allele. In addition, the MST shows that the Tunisian clusters are most closely related to countries in geographical proximity, with 150 discriminating alleles to Egypt, 245 to Algeria, and 266 to Morocco (Figure 2 and Figure 3). Of the thirty-six analyzed strains, five did not belong to any cluster, with one of them being very closely related to the proposed Tunisia Cluster 1. One of these isolates appears to be most closely related to single isolates from Morocco and Algeria. Interestingly, with less than 30 discriminating alleles, isolates from an outbreak in Austria cluster very closely to the proposed Tunisia Cluster 2 and three other strains from this sample set which could not be allocated to a cluster.

### 3.3. Identification of AMR and Virulence Genes from WGS Data

All the Tunisian strains presented the same virulence and AMR genes as the reference strains of biovars 1 and 3 (Table 4). For example, type IV secretion system genes (e.g, *virB*, *ricA* and *bsp*), immune modulation (*lpx*) and adhesins (*big*) were in every strain detected. Two genes encoding AMR were detected in all strains: *mprF* (multiple peptide resistance factors), which plays an essential role in resistance to cationic antibiotics such as gentamicin, and *bep G*, *F*, *C*, *E* and *D* genes (BPE membrane efflux protein), which can increase resistance to some antibiotic compounds such as tetracyclin, doxycyclin, chloramphenicol and ciprofloxacin.

## 4. Discussion

This study is one of the few Tunisian studies investigating human *Brucella* strains. Brucellosis is a zoonosis of worldwide distribution that is endemic in the Mediterranean basin, especially in the Northern African countries [28]. Despite the important animal reservoir for *Brucella* species in Tunisia, the epidemiological situation of human brucellosis was not known in Tunisia until the 1980s, particularly because of the misdiagnosis and the under-declaration of the disease [29]. Since the beginning of the 1990s, several outbreaks have occurred, such as the Gafsa (southern Tunisia) outbreak in 1991, with more than 400 human cases posing a serious public health threat. When eradication and control measures for both human and animal brucellosis were conducted, in Tunisia, the overall incidence for human brucellosis ranged between 2.9 and 4.5 per 100.000 inhabitants in 2015 and 2023, respectively. This could explain the significant number of cases recorded in 2016 and 2017 [30]. Despite the implemented programs, brucellosis is still occurring in Tunisia, with a variable annual incidence. As a chronic disease, it is of particular importance for public health, with indirect economic impacts such as reducing the work force and decreasing livestock reproduction and their associated products [5,31].

In this study, Tunisian human Brucella strains were clearly identified and characterized. A good and precise diagnosis with an exact identification of the bacterial species is essential to knowing the disease incidence in a region, to planning epidemiological studies, and to controlling eradication programs. Our results support the previous finding that human brucellosis can be a common public health problem in a large number of cities in Tunisia [32]. A comprehensive picture of the epidemiology and genetic diversity of *B. melitensis* in Tunisia was provided.

Tunisian *B. melitensis* strains presented an elevated genome similarity and shared almost all virulence- and AMR-related genes, even the same genes as the considered reference strains. *Brucella* species are well-known to present almost identical genomes in comparison with other bacterial species [25].

One of the strengths of this study lies in the analysis of database data on Human *B. melitensis* strains; this approach allows a more comprehensive understanding of the local situation regarding human brucellosis in Tunisia. However, a limitation of this study is the number of strains, emphasizing the need for larger datasets for more accurate future research.

Our phylogenetic analysis of *B. melitensis* followed similar phylogeographical patterns, and two main phylogenetic clusters were highlighted, with Tunisian Cluster 1 being more clearly defined (25 isolates) by the number of isolates than Tunisian Cluster 2 (6 isolates). Isolates from both populations were found essentially to circulate in the north and the north west of Tunisia. The overall distribution in Tunisia could not be assessed due to the bias of the catchment area of the two hospitals involved in the sampling. Notably, in 2016 and 2018, two important outbreaks of human brucellosis were observed in the south of Tunisia [30]. The two main branches closely related to Cluster 1 were linked to strains from Italy (the closest) and Egypt. The history of brucellosis spread in the Mediterranean basin could be explained by the strong trade connections between Italy and North Africa, which lasted from the time of the Roman Empire to the Middle Ages and continued in the last century during the period of modern colonialism [25]. Commercial activities between both regions could clarify the circulating *Brucella* species between African countries and the Northern Mediterranean region [29]. In this study, the Austrian isolates belonged to a branch including Tunisian Cluster 2 and were grouped together with Italian clusters, suggesting the hypothesis that both Italian and Austrian isolates were imported from Tunisia. This is especially supported by Figure 2, where a smaller Italian branch is calculated to originate from the strains identified in this study. A relationship between these countries was previously described in an Austrian study, which showed that *B. melitensis* isolates collected between 2005 and 2015 were closely related to a branch including strains from Egypt and Italy [30]. The studies characterizing the population of *B. melitensis* using WGS remain limited to some Mediterranean countries of the Maghreb region (Libya, Algeria, Morocco) [1]; we included recently published WGS data from Tunisia to expand the phylogeny and provide a comprehensive analysis.

To date, the few reported African Brucella genomes and the lack of *B. melitensis* genomes from other European countries within the Mediterranean region prevents us from knowing the real history of brucellosis in the region [28].

The implementation of high-throughput WGS to identify the AMR- and virulence-associated genes in *Brucella* isolates revealed no apparent difference in their distribution between *B. abortus* and *B. melitensis* strains or isolates from different hosts [33]. Investigations on antibiotic susceptibility and the updating of breakpoints are required. Investigations into resistance and virulence mechanisms at the proteomic and transcriptomic levels have to be considered in future research.

In conclusion, this study has helped to fill one of the gaps regarding the phylogenetic nature of the strains circulating in Tunisia in relation to its neighboring countries. This is an important step towards understanding the evolution of *Brucella melitensis* in this region. However, more data are needed from southern Tunisia and its neighboring countries to complete the picture and allow a more confident interpretation of the phylogenetic relationships presented today. On the other hand, knowing *Brucella* strains’ circulation encourages the government to strengthen control measures in the affected regions and regarding the borders of the country.

## Figures and Tables

**Table 1:** Oligonucleotide primers used in PCR assay.

*Gene*	*Primers*	Sequence (5′–3′)	*Localization*
***mazG***	*mazG-F*	GGATCTGATCGTAGCGACGGA	***3651–3671***
	*mazG-R*	CGTCCAATGTCTCACTGGAAAA	***3751–3772***
	Bru-mazG-TM-Multi	**FAM**-TGCCTTACATGGGCGAACTCGAACGT-**BHQ-1**	***3677–3703***
***IS711***	BruIS-F	GCCATCAGATTGAATGCTTTTTTAAC	***701–726***
	BruIS-R	AACCAGATCATAGCGCATGCG	***801–821***
	Bru-IS-TM-Multi	**Cy5**-CGCTGCGATGCGAGAAAACATTGACC-**BHQ-2**	***752–777***
***KoMa2***	KoMa-For	GGTGATGCCGCATTATTACTAGG	***198–220***
	KoMa-Rev	GGTATTAGCAGTCGCAGGCTT	***336–316***
	KoMa-TM	**HEX**-TTCTTGCTTGAGGATCTGTCGTGGATCG-**BHQ-2**	***224–251***

**Table 2:** Amplification program for real-time Polymerase Chain Reaction (RT-PCR).

**Temperatures [°C]**	**Time [s]**	**Cycles**	**Steps**
95	600		Denaturing
95	15	×40	Annealing Elongation + Detection
60	60		

**Table 3:** Collection of *Brucella* strains isolated from human blood and molecular identification.

Isolate	Accesion	Year	Hospital	Origin	Age (Years)	Gender	Clinical Form	cgMLST
A-1659-R23	SAMN38851271	2016	Rabta	Béja	37	M	Bacteremia	
A-1659-H1	SAMN38851272	2017	CNH	Ben Arous	45	F	Bacteremia	
A-1659-H3	SAMN38851273	2017	CNH	Tunis	42	M	Bacteremia	
A-1659-H4	SAMN38851274	2017	CNH	Siliana	37	F	Bacteremia	
A-1659-R1	SAMN38851275	2017	Rabta	Tunis	47	M	Bacteremia	
A-1659-R3	SAMN38851276	2017	Rabta	Tunis	55	M	Bacteremia	
A-1659-R4	SAMN38851277	2017	Rabta	Zaghouen	37	F	Bacteremia	
A-1659-R12	SAMN38851278	2017	Rabta	Tunis	41	M	Bacteremia	
A-1659-R13	SAMN38851279	2017	Rabta	Nabeul	28	F	Bacteremia	
A-1659-R15	SAMN38851280	2017	Rabta	Tunis	39	M	Bacteremia	
A-1659-R16	SAMN38851281	2017	Rabta	Béja	59	M	Bacteremia	
A-1659-R18	SAMN38851282	2017	Rabta	Tunis	37	M	Bacteremia	
A-1659-R20	SAMN38851283	2017	Rabta	Tunis	51	M	Bacteremia	Tunisia
A-1659-R27	SAMN38851284	2017	Rabta	Béja	22	M	Bacteremia	Cluster 1
A-1659-R31	SAMN38851285	2017	Rabta	Ben Arous *	27	M	Bacteremia	
A-1659-R33	SAMN38851286	2017	Rabta	Mannouba	25	M	Bacteremia **	
A-1659-R35	SAMN38851287	2017	Rabta	Ben Arous	21	M	Bacteremia	
A-1659-R37	SAMN38851288	2017	Rabta	Tunis	20	F	Bacteremia	
A-1659-R38	SAMN38851289	2017	Rabta	Bizete	30	M	Bacteremia	
A-1659-R39	SAMN38851290	2017	Rabta	Tunis	68	M	Bacteremia	distant
A-1659-H2	SAMN38851291	2017	CNH	Béja	35	F	Bacteremia	
A-1659-R14	SAMN38851292	2017	Rabta	Bizerte	48	M	Bacteremia	
A-1659-R26	SAMN38851293	2017	Rabta	Tunis	63	M	Bacteremia	
A-1659-R28	SAMN38851294	2017	Rabta	Tunis	33	M	Bacteremia	
A-1659-R30	SAMN38851295	2017	Rabta	Tunis	63	M	Bacteremia	
A-1659-R36	SAMN38851296	2017	Rabta	Tunis	47	M	Bacteremia	
A-1659-H5	SAMN38851297	2017	CNH	Tunis	23	M	Bacteremia	distant
A-1659-R2	SAMN38851298	2017	Rabta	Seliana	59	M	Spondilodiscitis	
A-1659-R6	SAMN38851299	2017	Rabta	Béja	71	M	Bacteremia	
A-1659-R11	SAMN38851300	2017	Rabta	Tunis	21	M	Bacteremia	Tunisia
A-1659-R17	SAMN38851301	2016	Rabta	Béja	22	M	Bacteremia	Cluster 2
A-1659-R19	SAMN38851302	2017	Rabta	Tunis	25	M	Bacteremia	
A-1659-R25	SAMN38851303	2016	Rabta	Ben Arous	16	M	Bacteremia	
A-1659-P1	SAMN38851304	2020	CNH	Tunis	37	F	Bacteremia	distant
A-1659-R24	SAMN38851305	2016	Rabta	Béja	68	M	Bacteremia	distant
A-1659-R32	SAMN38851306	2016	Rabta	Bizerte	26	M	Bacteremia	distant

Accession: NCBI accession number; Recult.: recultivation; M: Male; F: female; Rabta: Rabta Hospital; CNH: Charles Nicole Hospital. (*) indicates contamination in Saudi Arabia, (**) indicates Orchitis.

**Table 4:** Virulence- and antimicrobial resistance (AMR)-related genes detected * in all Tunisian strains as well as in *Brucella melitensis* bv. 1 16M and *B. melitensis* bv. 3 Ether.

Type	Virulence	AMR-Related
Gene	*acpXL*, *bigA*, *bigB*, *bmaB*/*omaA*, *bmaC*, BPE005, BPE043, BPE123, BPE275, *bspA*, *bspB*, *bspC*, *bspE*, *bspF*, *bspL*, *btpA*, *btpB*, *bvrR*, bvrS, *cgs*, *fabZ*, *gmd*, *htrB*, *kdsA*, *kdsB*, *lpsA*, *lpsB*/*lpcC*, *lpxA*, *lpxB, lpxC, lpxD*, *lpxE*, *lpxK*, *manAoAg*, *manBcore*, *manCcore*, *manCoAg*, *per*, *pgm*, *pmm*, *ricA*, *sepA*, *vceA*, *vceC*, *virB1*, *virB10*, *virB11*, *virB12*, *virB2*, *virB3*, *virB4*, *virB5*, *virB6*, *virB7*, *virB8*, *virB9*, *waaA*/*kdtA*, *wbdA*, *wbkA*, *wbkB*, *wbkC*, *wboA*, *wbpL*, *wbpZ*, *wzm*, *wzt*	*bepC*, *bepD*, *bepE*, *bepF*, *bepG*, *mprF*

(*) with a coverage and identity > 98%.

## Data Availability

The original contributions presented in this study are included in the article. Further inquiries can be directed to the corresponding author.
